# The History and Capabilities of Herbal Simples

**Published:** 1891-07-04

**Authors:** W. T. Fernie


### The History and Capabilities of Herbal Simples.

By W. T. Fernie, M.D.

### XXXIV.—FENNEL.

All of us who have eaten boiled mackerel know the pleasant taste of Fennel in the sauce which is its invariable accompaniment. This culinary condiment is made with sweet fennel, cultivated in our gardens; which is a variety of the wild fennel growing commonly in England, especially on chalky cliffs near the sea. Provincially it is called "finkel," and botanically it is the anethum fœniculum or "little hay" of the Romans, and the marathron of the Greeks. It belongs to the umbelliferous order of plants, but differs from the rest of the tribe in possessing bright yellow flowers. The whole plant has a fragrant odour, with a warm, aromatic taste; and the old Greeks esteemed it highly for promoting the secretion of milk in nursing mothers. Hippocrates and Dioscorides ordered its use for this purpose, "*mais il en porte le gout*," says a modern medical authority. Macer alleged that the use of fennel was first taught to man by serpents.

"Hac mansâ serpens oculos caligine purgat;
Indeque compertum est humanis posse mederi
Illam luminibus; atque experiendo probatum est."

"By eating herb of fennel for the eyes
A cure for blindness had the serpent wise;
Man tried the plant, and, trusting that his sight
Might thus be healed, rejoiced to find him right."

Pliny repeats the assertion that the ophidia when they cast their skins have recourse to this plant for restoring their sight. And in allusion to the same belief Shakespeare makes the sister of Laertes say to the king in "Hamlet," "There's Fennel for you, and Columbines," when wishing to quicken the royal conscience. Further, in the play of "Henry IV.," Falstaff says of Poins, "He plays at quoits well, and eats congor and fennel." In the Elizabethan age this herb was quoted as an emblem of flattery—"Little things," it was then said, "catch light minds; and fancie is a worm that feedeth first upon fennel." The Italians eat blanched stalks of the sweet fennel as a salad, with vinegar, oil, and pepper. In Germany, the seeds are added to bread as a condiment, much as we put caraways in our common cakes. The leaves are eaten raw with pickled fish, to correct its oily indigestibility. Chemically, the fennel plant furnishes an aromatic volatile oil, a fixed fatty principle, sugar, and some starch in the root; also a resinous bitter extract. A hot infusion made by pouring half a pint of boiling water on a teaspoonful of the bruised seeds will relieve flatulent belly-ache in the infant if given in teaspoonful doses, sweetened with sugar, and will prove an active remedy for promoting the monthly courses with regularity if taken at the time in doses of a wineglassful thrice a day. Gerarde says, "the green leaves of the fennel eaten, or the seed made into a ptisan, and drunk, do fill women's brests with milke; also, the seed, if drunk, asswageath the wambling of the stomacke, and breaketh the winde." The essential oil corresponds in composition to that of Anise, but contains a special camphoraceous body of its own; whilst its vapour cause the tears and the saliva to flow. For griping of the bowels with distension, from two to six drops of the essential oil may be taken on a lump of sugar. A syrup prepared from the expressed juice was formerly given in chronic coughs. Some authors have averred that serpents wax young again by eating of this herb, wherefore the use of it is very meet for aged folk. W. Coles teaches in "Nature's Paradise" that "both the leaves, seeds, and roots are much used in drinks and broths for those that are grown fat, to abate their unwieldinesse, and make them more gaunt and lank." The ancient Greek name—"marathron"—of the plant, from *maraino*, to grow thin, probably embodied the same notion. "In warm climates," said Matthiolus, "the stems are cut, and there exudes a resinous liquid which is collected under the name of fennel gum." A modern distilled water is now obtained from the herb which is a capital carminative for babies, and an admirable collyrium for weak eyes. The bruised plant applied outside, speedily relieves toothache or earache. The flower, surrounded by its four leaves, is called in the South of England "Devil in a bush." An old proverb which at one time obtained with us, and is still believed in New England, says that "sowing fennel is sowing sorrow."

